# Effectiveness of daily versus non-daily granulocyte colony-stimulating factors in patients with solid tumours undergoing chemotherapy: a multivariate analysis of data from current practice

**DOI:** 10.1111/ecc.12043

**Published:** 2013-01-18

**Authors:** D Almenar Cubells, C Bosch Roig, E Jiménez Orozco, R Álvarez, JM Cuervo, N Díaz Fernández, AB Sánchez Heras, A Galán Brotons, V Giner Marco, M Codes M De Villena, ON BEHALF OF LEARN II STUDY GROUP

**Affiliations:** Medical Oncology Service, Hospital Universitario Dr PesetValencia; Clinical Management Unit of Medical Oncology, Hospital General de JerezJerez de la Frontera; Medical Oncology Service, Hospital Virgen de la SaludToledo; Medical Oncology Service, Hospital San Juan de Dios de BurgosBurgos; Medical Oncology Service, Hospital Clínico Universitario San JuanSant Joan d'Alacant; Medical Oncology Service, Hospital General de ElcheElche; Medical Oncology Service, Hospital de SaguntoSagunto; Clinical Management Unit of Medical Oncology, Hospital Virgen MacarenaSevilla, Spain

**Keywords:** neutropenia, granulocyte-colony stimulating factors, multi-cycle chemotherapy, pegfilgrastim, filgrastim, lenograstim

## Abstract

We conducted a multicentre, retrospective, observational study including patients with solid tumours (excluding breast cancer) that received granulocyte colony-stimulating factors (G-CSF) and chemotherapy. We investigated the effectiveness of daily vs. non-daily G-CSFs (pegfilgrastim) adjusting by potential confounders. The study included 391 patients (211 daily G-CSF; 180 pegfilgrastim), from whom 47.3% received primary prophylaxis (PP) (57.8% pegfilgrastim), 26.3% secondary prophylaxis (SP: initiation after cycle 1 and no reactive treatment in any cycle) (51.5% pegfilgrastim) and 26.3% reactive treatment (19.4% pegfilgrastim). Only 42.2% of patients with daily G-CSF and 46.2% with pegfilgrastim initiated prophylaxis within 72 h after chemotherapy, and only 10.5% of patients with daily G-CSF received it for ≥7 days. In the multivariate models, daily G-CSF was associated with higher risk of grade 3-4 neutropenia (G3-4N) vs. pegfilgrastim [odds ratio (OR): 1.73, 95% confidence interval (CI): 1.004–2.97]. Relative to SP, PP protected against G3-4N (OR for SP vs. PP: 6.0, 95%CI: 3.2–11.4) and febrile neutropenia (OR: 3.1, 95%CI: 1.1–8.8), and was associated to less chemotherapy dose delays and reductions (OR for relative dose intensity <85% for SP vs. PP: 3.1, 95%CI: 1.7–5.4) and higher response rate (OR: 2.1, 95%CI: 1.2–3.7). Data suggest that pegfilgrastim, compared with a daily G-CSF, and PP, compared with SP, could be more effective in preventing neutropenia and its related events in the clinical practice.

## Introduction

Granulocyte colony-stimulating factors (G-CSF) are cytokines that regulate the proliferation, differentiation, and survival of haematopoietic stem cell precursors and mature peripheral effector cells. The preventive use of G-CSF in cancer patients undergoing chemotherapy according to current international guidelines can help overcome the frequent problem of chemotherapy-related myelotoxicity (Crawford *et al*. 1991, 2005; Messori *et al*. 1996; Aapro *et al*. 2006; Gabrilove 2006; Kuderer *et al*. 2006, 2007; NCCN 2012). The occurrence of this complication is strictly related to the characteristics of the employed chemotherapy regimens, including the interval between cycles.

Neutropenia and febrile neutropenia not only can compromise the safety of the patient and cause unplanned hospitalisations, but also can induce dose reduction in a large percentage of patients (Lyman 2006). The role of chemotherapy and the maintenance of its doses and schedules are of great importance, particularly in the so-called chemosensitive tumours (Marangolo *et al*. 2006). Haematological toxicity remains the main cause of dose reductions or delays, and the reduction of dose intensity can potentially worsen clinical outcome in some cases.

Daily G-CSFs, such as filgrastim or lenograstim, should be administered daily until the expected neutrophil nadir is passed and the neutrophil count has recovered to the normal range, which usually requires up to 2 weeks of treatment. In clinical trials where daily G-CSFs were continued until either the neutrophil count reached ≥10 × 10^9^/L or for up to 14 days, whichever occurred first, the mean duration of G-CSF prophylaxis was up to 10–11 days (Dale *et al*. 1993; Muhonen *et al*. 1996; Bishop *et al*. 2000; Holmes *et al*. 2002b; Green *et al*. 2003; Siena *et al*. 2003). However, several observational studies have reported that in most patients treated in the clinical practice, duration of prophylaxis with daily G-CSFs is less than 7 days, and that a short duration of daily G-CSF treatment is associated with worse neutropenia-related clinical outcomes (Weycker *et al*. 2006; Morrison *et al*. 2007). From these studies, it is derived that an adequate use of G-CSF prophylaxis is crucial in chemotherapy regimens in which the maintenance of dose intensity or increase of dose-dense is the most important tool for outcome. The day of initiation after chemotherapy delivery is also variable, and an early use of myeloid growth factors, before the neutrophil count is too low, is crucial for ensuring their efficacy (Shochat & Rom-Kedar 2008).

A pegylated formulation of filgrastim, pegfilgrastim, which is administered once per cycle, may play an important role in reducing the duration and incidence of haematological toxicities by ensuring an adequate G-CSF administration (Holmes *et al*. 2002a; Grigg *et al*. 2003; Schippinger *et al*. 2006; Pinto *et al*. 2007; Eldar-Lissai *et al*. 2008). The LEARN I study, conducted in 2003, was a multicentre, retrospective, observational study in Spain comparing patterns of use of daily G-CSF and pegfilgrastim, and neutropenia-related outcomes in adults with non-myeloid malignancies receiving myelosuppressive chemotherapy (Almenar *et al*. 2009). In that study, chemotherapy-induced neutropenia-related complications were less frequent in patients receiving pegfilgrastim, and the median number of injections per cycle in patients treated with filgrastim was 6 in the first cycle and 5 in the subsequent cycles or in reactive use.

The present observational study was designed to provide updated information about patterns of use of G-CSFs in the clinical practice of Spanish oncology services and to compare neutropenia-related outcomes in patients treated with a daily G-CSF compared with patients treated with a non-daily G-CSF (pegfilgrastim), after adjusting by potential confounders.

## Patients and methods

**Figure 1:**
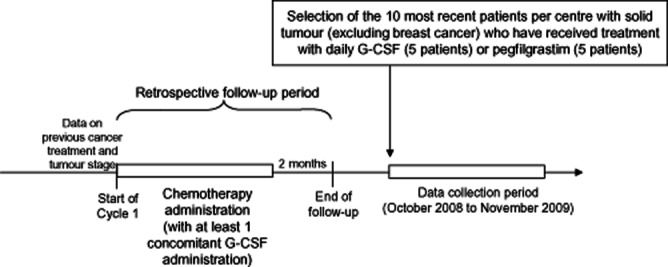
Study scheme. G-CSF, granulocyte colony-stimulating factors.

We conducted a multicentre, retrospective, observational, two-cohort study that included adult patients (≥18 years) with solid tumour (excluding breast cancer) who had undergone chemotherapy with at least one concomitant G-CSF (daily or non-daily) administration more than 2 months ago. Patients who had participated in a clinical trial during the retrospective follow-up period (see Fig. 1) or who had insufficient data in the patient file regarding the primary outcome were excluded. Fifty medical oncology services of public and private hospitals geographically distributed across Spain were invited to participate in the study, and 68% (*n* = 34) agreed to participate. To reduce biases, each investigator was asked to review the patient files of the most recent five patients treated with a daily G-CSF and the most recent five patients treated with pegfilgrastim in the centre. Data collection period ranged from October 2008 to November 2009. The protocol was approved by an independent ethics committee.

The primary outcome measure was the percentage of patients with grade 3-4 (G3-4) neutropenia over the follow-up period, defined as absolute neutrophil count (ANC) < 1.0 × 10^9^/L. Secondary outcome measures included incidence of febrile neutropenia (FN) (defined as ANC < 0.5 × 10^9^/L and fever ≥38°C within the same day) over the follow-up period, percentage of patients with chemotherapy dose delays (defined as a delay of >3 days in any cycle) or reductions (defined as <85% of planned chemotherapy dose for any agent in the regimen in any cycle), percentage of patients with chemotherapy dose intensity ≥85% (defined as ≥85% of planned chemotherapy dose for all agents in the regimen and ≤3 days dose delay) over all cycles, number and duration of FN-related hospitalisations and response to chemotherapy treatment according to physician's criterion [complete response, partial response or non-response (stable or progressive disease)]. Data on demographic and clinical characteristics, baseline serum biochemistry, chemotherapy and G-CSF administration, adverse effects related to G-CSFs, hospitalisations due to other reasons and use of antibiotics during the follow-up period were also collected. Type of G-CSF use was recorded by the investigator in the case report form and defined as primary prophylaxis (G-CSF use in the first cycle before a documented G3-4 neutropenia occurred), secondary prophylaxis (incorporating G-CSF prophylaxis in cycles other than the first before a documented G3-4 neutropenia occurred and no reactive treatment in any cycle) or treatment (G-CSF use as reactive treatment for neutropenia).

### Statistical analyses

Results are given as mean and standard deviation (or median and quartiles) for continuous measures and as numbers and percentages for categorical measures. Data were compared between the two cohorts (daily G-CSF and pegfilgrastim) and between subgroups defined by patterns of use of G-CSF and number of days of daily G-CSF treatment using Student's *t*-tests or Mann–Whitney *U*-tests for continuous variables and chi-squared tests for categorical variables. Multivariate logistic regression models were constructed to examine the effect of type of G-CSF used (daily or non-daily) on five study outcomes [G3-4 neutropenia, febrile neutropenia, hospitalisation due to febrile neutropenia, chemotherapy dose intensity <85% and response to chemotherapy (partial or complete)] after adjusting by possible confounders. Variables were selected using a stepwise technique (using *P* ≤ 0.20). When collinearity was observed, the variable less strongly associated with the outcome of interest was excluded from the model. spss version 17.0 (SPSS Inc, Chicago, Illinois, USA) was used for all analyses.

## Results

### Characteristics of patients who received daily G-CSF or pegfilgrastim

The investigators of the 34 participating centres reviewed a total of 441 patient files. Fifty patients were excluded for having received more than one type of G-CSF. Thus, the study included 391 patients treated between January 1999 and December 2008, 211 who had received a daily G-CSF (196 with filgrastim and 15 with lenograstim) and 180 who had received a non-daily G-CSF (pegfilgrastim). Table 1 shows the main clinical characteristics, patterns of use of G-CSF and chemotherapy administration in the two study cohorts. Patients who received a daily G-CSF were older, had more gastrointestinal tumour type and were treated with less cytotoxic antibiotics and completed a higher number of chemotherapy cycles. No significant differences were found in the other clinical or demographic characteristics, including tumour stage, previous cancer treatment or baseline serum biochemistry. Most patients in both groups had advanced stage (III–IV) tumours (79.3%).

**Table 1:** Main characteristics of the two study cohorts (daily G-CSF and pegfilgrastim) and chemotherapy received

	Daily G-CSF (*n* = 211)	Pegfilgrastim (*n* = 180)	*P*-value
Age (years), mean (SD)	61.7 (12.2)	57.9 (13.7)	0.005
≥65 years, *n* (%)	98 (46.4)	57 (31.8)	0.003
Men, *n* (%)	139 (65.9)	124 (68.9)	0.527
Tumour type, *n* (%)			0.006
Lung	55 (26.1)	48 (26.7)	
Gastrointestinal	59 (28.0)	28 (15.6)	
Gynaecologic	34 (16.1)	22 (12.2)	
Head and neck	25 (11.8)	27 (15.0)	
Other*	38 (18.0)	55 (30.5)	
Stage, *n* (%)			0.435
I	19 (9.0)	9 (5.0)	
II	22 (10.4)	22 (12.2)	
III	61 (28.9)	50 (27.8)	
IV	104 (49.3)	95 (52.8)	
Unknown	5 (2.4)	4 (2.2)	
Previous cancer treatment, *n* (%)	72 (34.1)	59 (33)	0.809
Previous chemotherapy	62 (29.4)	49 (27.4)	0.661
Previous radiotherapy	37 (17.5)	27 (15.1)	0.515
Chemotherapy regimen, *n* (%)			
Platinum agent	162 (76.8)	146 (81.1)	0.296
Taxane	75 (35.5)	72 (40.0)	0.365
Mustard analogues	7 (3.3)	11 (6.1)	0.189
Pyrimidine analogues	92 (43.6)	72 (40.0)	0.472
Cytotoxic antibiotics	11 (5.2)	19 (10.6)	0.048
Number of completed cycles, mean (SD)	5.2 (2.6)	4.6 (2.3)	0.010
G-CSF use, *n* (%)			<0.0001
Primary prophylaxis	78 (37.0)	107 (59.5)	
Secondary prophylaxis	50 (23.7)	53 (29.4)	
Reactive use	83 (39.3)	20 (11.1)	
Day of initiation of G-CSF after last chemotherapy dose *(only in patients with primary of secondary prophylaxis)*, *n* (%)			0.490
Within 72 h after CT	54 (42.2)	74 (46.2)	
>72 h after CT	74 (57.8)	86 (53.8)	
Dose of G-CSF, median (range)	300 (263–480)	6.0 (6.0–6.0)	–
Days of administration of G-CSF, median (range)	5 (1–18)	1 (1–1)	–
Baseline serum biochemistry, mean (SD)			
Haemoglobin, g/dL	12.5 (2.0)	12.7 (1.8)	0.249
Leucocyte count, ×10^9^/L	7.2 (3.3)	7.7 (3.5)	0.139
Neutrophil count, ×10^9^/L	4.7 (2.9)	4.9 (3.0)	0.478
Lymphocyte count, ×10^9^/L	2.5 (5.4)	3.2 (7.0)	0.256
Monocyte count, ×10^9^/L	0.4 (0.2)	0.4 (0.3)	0.545
Platelet count, ×10^9^/L	273 (123)	281 (126)	0.556
Bilirrubin, mg/dL	0.6 (0.5)	0.5 (0.3)	0.432
Creatinine, mg/dL	0.9 (0.3)	0.9 (0.4)	0.861
LDH, U/L	303 (382)	336 (357)	0.460
GOT, U/L	26.6 (15.9)	26.3 (17.8)	0.861
GPT, U/L	28.1 (18.7)	31.1 (25.8)	0.259
Albumin, g/dL	4 (0.5)	4 (0.6)	0.788

* Other tumour types: kidney (10 and 13 patients, respectively, in daily G-CSF and pegfilgrastim groups); testicular (7 and 15 patients); prostate (5 and 6 patients); mediastinum, peritoneum or retroperitoneum (4 and 5 patients); pancreas (6 and 2 patients); soft tissue (5 and 10 patients); bone (0 and 3 patients), central nervous system (1 and 1 patient).

G-CSF, granulocyte colony-stimulating factors; GOT, glutamyl oxaloacetic transaminase; GPT, glutamyl pyruvic transaminase; LDH, lactate-dehydrogenase.

**Figure 2:**
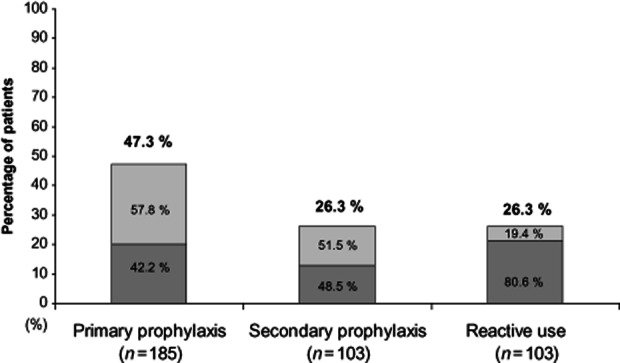
Patterns of use of G-CSF treatment in the study sample. 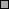, Pegfilgrastim; 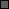, Daily G-CSF. G-CSF, granulocyte colony-stimulating factors.

Overall, 47.3% of patients received primary prophylaxis (57.8% with pegfilgrastim), 26.3% received secondary prophylaxis (51.5% with pegfilgrastim) and 26.3% received reactive treatment (19.4% with pegfilgrastim) (Fig. 2). Patients selected for primary prophylaxis, compared with patients who received secondary prophylaxis, were characterised by (Table 2): higher percentage of stage IV tumours; less number of chemotherapy cycles; lower frequency of gastrointestinal and gynaecologic cancer type and higher frequency of soft tissue or testicular tumours; higher use of taxane-, mustard analogue- and cytotoxic antibiotics-containing regimens; higher mean baseline leucocyte count, neutrophil count and GOT (glutamyl oxaloacetic transaminase) levels. They also received a median of five injections of daily G-CSF, compared with only four in patients with SP (secondary prophylaxis). No differences were observed in the other clinical or chemotherapy characteristics.

**Table 2:** Main characteristics and administered chemotherapy in subgroups of patients defined by pattern of use of G-CSF (primary prophylaxis, secondary prophylaxis or reactive use)

Variables	Primary prophylaxis (*n* = 185)	Secondary prophylaxis (*n* = 103)	Reactive use (*n* = 103)	*P*-value (*PP* vs. *SP*)
Age (years), mean (SD)	59.8 (13.3)	60.2 (11.6)	60.1 (13.9)	0.785
≥65 years, *n* (%)	72 (38.9)	37 (35.9)	46 (45.1)	0.615
Men, *n* (%)	130 (70.3)	66 (64.1)	67 (65.0)	0.280
Tumour type, *n* (%)				0.010
Lung	49 (26.5)	31 (30.1)	23 (22.3)	
Gastrointestinal	28 (15.1)	25 (24.3)	34 (33.0)	
Gynaecologic	26 (14.1)	21 (20.4)	9 (8.8)	
Head and neck	30 (16.2)	13 (12.6)	9 (8.8)	
Soft tissue	12 (6.5)	1 (1.0)	2 (2.0)	
Testicular	12 (6.5)	1 (1.0)	9 (8.8)	
Prostate	7 (3.8)	0 (0)	4 (3.9)	
Other	21 (11.4)	11 (10.7)	13 (12.6)	
Stage, *n* (%)				0.037
I	11 (5.9)	9 (8.7)	8 (7.8)	
II	12 (6.5)	16 (15.6)	16 (15.6)	
III	50 (27.0)	28 (27.2)	33 (32.0)	
IV	109 (58.8)	47 (45.6)	43 (41.7)	
Unknown	3 (1.6)	3 (2.9)	3 (2.9)	
Previous cancer treatment, *n* (%)	60 (32.4)	36 (35.0)	35 (34.0)	0.664
Previous chemotherapy	49 (26.5)	30 (29.1)	32 (31.4)	0.630
Previous radiotherapy	27 (14.6)	20 (19.4)	17 (16.7)	0.288
Chemotherapy regimen, *n* (%)				
Platinum agent	144 (77.8)	90 (87.4)	74 (71.8)	0.047
Taxane	91 (49.2)	29 (28.2)	27 (26.2)	0.001
Mustard analogues	14 (7.6)	1 (1.0)	3 (2.9)	0.016
Pyrimidine analogues	68 (36.8)	44 (42.7)	52 (50.5)	0.320
Cytotoxic antibiotics	17 (9.2)	2 (1.9)	11 (10.7)	0.018
Number of completed cycles, mean (SD)	4.2 (2.1)	5.7 (2.3)	5.3 (2.8)	<0.001
G-CSF use, *n* (%)				0.296
Daily G-CSF	78 (42.2)	50 (48.5)	83 (80.6)	
Non-daily G-CSF	107 (57.8)	53 (51.5)	20 (19.4)	
Day of initiation of G-CSF after last chemotherapy dose *(only in patients with primary or secondary prophylaxis)*, *n* (%)				0.147
Within 72 h after CT	69 (37.3)	47 (46.1)	–	
>72 h after CT	116 (62.7)	55 (53.9)	–	
Dose of G-CSF, median (range)	300 (263–480)	300 (263–480)	300 (300–480)	0.300
Days of administration of G-CSF, median (range) *(only in patients with daily G-CSF)*	5 (1–11.2)	4 (1–9)	4.9 (1–14)	0.002
Baseline serum biochemistry, mean (SD)				
Haemoglobin, g/dL	12.6 (1.9)	12.8 (2.1)	12.4 (1.7)	0.366
Leucocyte count, ×10^9^/L	8.3 (3.5)	6.8 (3.4)	6.6 (2.8)	0.000
Neutrophil count, ×10^9^/L	5.4 (3)	4.1 (3.1)	4.2 (2.4)	0.001
Lymphocyte count, ×10^9^/L	2.6 (4.9)	3.7 (8.5)	2.2 (4.6)	0.224
Monocyte count, ×10^9^/L	0.4 (0.3)	0.5 (0.2)	0.4 (0.2)	0.165
Platelet count, ×10^9^/L	289 (136)	279 (115)	253 (108)	0.552
Bilirrubin, mg/dL	0.6 (0.3)	0.6 (0.6)	0.5 (0.3)	0.900
Creatinine, mg/dL	0.9 (0.4)	0.9 (0.3)	1 (0.4)	0.158
LDH, U/L	307 (373)	361 (457)	290 (223)	0.356
GOT, U/L	29.2 (20.1)	23.5 (11.7)	24.5 (13.9)	0.023
GPT, U/L	32.3 (22.9)	28.7 (25.6)	25.2 (16.1)	0.300
Albumin, g/dL	4 (0.5)	4 (0.6)	4 (0.6)	0.480

G-CSF, granulocyte colony-stimulating factors; GOT, glutamyl oxaloacetic transaminase; GPT, glutamyl pyruvic transaminase; LDH, lactate-dehydrogenase; PP, primary prophylaxis; SP, secondary prophylaxis.

Only 42.2% and 46.2% of patients with daily-GSF and pegfilgrastim, respectively, initiated treatment within 72 h after chemotherapy. Also only 10.5% of patients with a daily G-CSF received it for ≥7 days (l4.3% received it for ≥6 days and 45.9% received it for ≥5 days).

### Neutropenia-related outcomes

**Figure 3:**
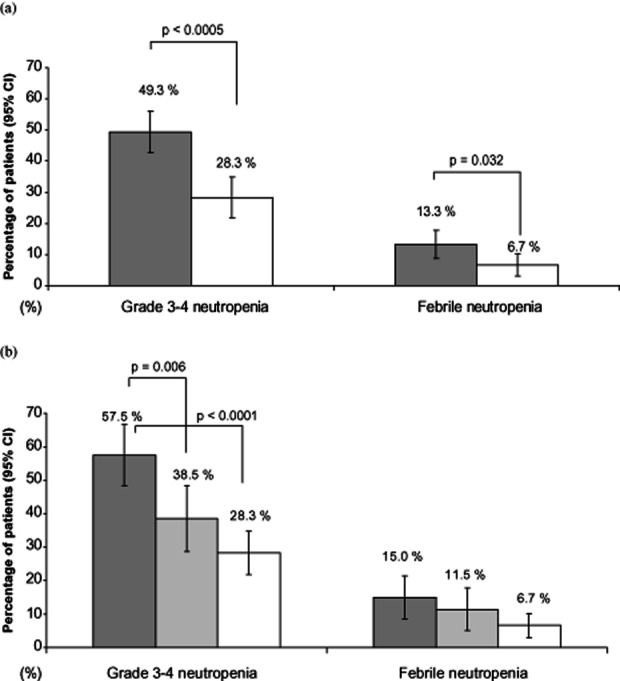
Incidence of severe neutropenia and febrile neutropenia in subgroups of patients receiving: (a) daily G-CSF or pegfilgrastim. 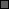, Daily G-CSF; 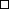, Pegfilgrastim. (b) daily G-CSF (<5 days or ≥5 days) or pegfilgrastim. 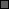, Daily G-CSF (<5 days); 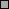, Daily G-CSF (≥5 days); 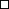, Pegfilgrastim. G-CSF, granulocyte colony-stimulating factors.

Table 3 shows neutropenia-related outcomes in patients who had received daily compared with pegfilgrastim. Patients with daily G-CSF suffered more G3-4 and febrile neutropenia (Fig. 3a), and more hospitalisations due to neutropenia, severe neutropenia or febrile neutropenia. They also received more antibiotics due to neutropenia. A trend toward more hospitalisation due to infection was observed, but differences were not statistically significant. In patients hospitalised due to neutropenia, the median number of hospital days was comparable for the two groups.

**Table 3:** Neutropenia-related outcomes, hospitalisations and antibiotics use in patients who received daily G-CSF or pegfilgrastim

Outcome	Daily G-CSF (*n* = 211)	Pegfilgrastim (*n* = 180)	
	
	*n* (%)		*P*-value
Grade 3-4 neutropenia, *n* (%)	104 (49.3)	51 (28.3)	<0.0005
Febrile neutropenia, *n* (%)	28 (13.3)	12 (6.7)	0.032
Any hospitalisation, *n* (%)	71 (33.6)	46 (25.6)	0.082
Hospitalisation due to neutropenia, *n* (%)	31 (14.7)	7 (3.9)	<0.0005
Length of hospital stay, median days (P25-P75)	9 (5–12)	8 (6–10)	0.821
Hospitalisation due to severe neutropenia, *n* (%)	26 (12.3)	6 (3.3)	0.001
Length of hospital stay, median days (P25-P75)	9 (6–12)	9 (6–10)	0.753
Hospitalisation due to febrile neutropenia, *n* (%)	23 (10.9)	5 (2.8)	0.002
Length of hospital stay, median days (P25-P75)	9 (6–15)	8 (6–10)	0.630
Hospitalisation due to infection, *n* (%)	12 (5.7)	4 (2.2)	0.085
Hospitalisation due to fever, *n* (%)	9 (4.3)	7 (3.9)	0.851
Hospitalisation due to pancitopenia, *n* (%)	6 (2.8)	2 (1.1)	0.400
Hospitalisation due to other haematologic toxicities, *n* (%)	6 (2.8)	2 (1.1)	0.267
Antibiotic use due to neutropenia, *n* (%)	50 (23.7)	17 (9.4)	0.002
Antibiotic use due to febrile neutropenia, *n* (%)	24 (11.4)	8 (4.4)	0.013

G-CSF, granulocyte colony-stimulating factors.

In the multivariate analysis, after adjusting for possible confounding factors, daily G-CSF was associated with a significantly higher risk of severe neutropenia vs. pegfilgrastim [odds ratio (OR): 1.73, 95% confidence interval (CI): 1.004–2.97, i.e. patients who received a daily G-CSF had a 73% higher probability of G3-4N] (Table 4). Other factors independently associated with severe neutropenia (G3-4N) were: low baseline neutrophil count (<1.5 × 10^9^/L), serum creatinine >1.5 g/dL, platinum-based chemotherapy, anthracycline-based chemotherapy and a high number of chemotherapy cycles. Relative to SP, PP (primary prophylaxis) with G-CSF protected against G3-4N.

**Table 4:** Independent predictors of grade 3-4 neutropenia occurrence in patients with solid tumours (excluding breast cancer) treated with G-CSF in the clinical practice (multivariate logistic regression model)

Predictors of grade 3-4 neutropenia	Odds ratio (OR)	95% CI for OR	*P*-value
Baseline creatinine >1.5 g/dL	4.82	1.44–16.06	0.011
Baseline neutrophil count <1.5 × 10^9^/L	5.92	2.12–16.52	0.001
Platinum-containing chemotherapy regimen	2.92	1.44–5.9	0.003
Cytotoxic antibiotics-containing chemotherapy regimen	3.32	1.17–9.43	0.024
Number of chemotherapy cycles (for each additional cycle)	1.16	1.03–1.29	0.010
Prophylactic G-CSF use (*ref. primary prophylaxis*)			<0.0005*
Secondary prophylaxis	6.03	3.2–11.36	<0.0005
Treatment	18.34	9.27–36.26	<0.0005
Daily G-CSF (*ref. pegfilgrastim*)	1.73	1.004–2.97	0.048

*n* = 391.

* *P*-value indicates the overall effect of the variable in the model.

Other variables tested and not included in the final model were: age, gender, stage, tumour type, previous chemotherapy, intention of treatment, taxane-containing chemotherapy regimen, mustard analogues-containing chemotherapy regimen; pyrimidine analogues-containing chemotherapy regimen, baseline lymphocyte count, baseline monocyte count, baseline platelet count, baseline bilirrubin, baseline LDH, baseline GOT, baseline GPT, baseline albumin, baseline haemoglobin, G-CSF use within 72 h after chemotherapy.

CI, confidence interval; G-CSF, granulocyte colony-stimulating factors; GOT, glutamyl oxaloacetic transaminase; GPT, glutamyl pyruvic transaminase; LDH, lactate-dehydrogenase; OR, odds ratio.

In the multivariate model for predicting FN, no significant effect was found for the type of G-CSF (OR: 1.1 for daily vs. non-daily, 95% CI: 0.47–2.6), but a higher probability of FN was observed in patients receiving SP (OR: 3.1, 95% CI: 1.1–8.8) or reactive treatment with G-CSF (OR: 11.3, 95% CI: 3.9–32.9), relative to PP.

Table 5 shows the multivariate model for predicting hospitalisation due febrile neutropenia. A trend towards a higher risk of hospitalisation in patients treated with daily vs. pegfilgrastim was observed, but the effect did not achieve statistical significance (*P* = 0.176).

**Table 5:** Independent predictors of hospitalisation due to febrile neutropenia in patients with solid tumours (excluding breast cancer) treated with G-CSF in the clinical practice (multivariate logistic regression model)

Predictors of hospitalisation due to febrile neutropenia	Odds ratio (OR)	95% CI for OR	*P*-value
Baseline creatinine >1.7 g/dL	8.54	1.67–43.75	0.010
Neoadjuvant or palliative chemotherapy *(ref. adjuvant CT)*	4.04	1.09–14.92	0.037
Mustard analogues-containing chemotherapy regimen	5.07	1.10–23.28	0.037
Head and neck cancer (*ref. all other tumour types*)	4.84	1.67–14.03	0.004
Prophylactic G-CSF use (*ref. primary prophylaxis*)			0.020*
Secondary prophylaxis	1.46	0.37–5.78	0.586
Treatment	4.97	1.50–16.42	0.009
Daily G-CSF (*ref. pegfilgrastim*)	2.23	0.70–7.12	0.176

*n* = 344.

* *P*-value indicates the overall effect of the variable in the model.

Other variables tested and not included in the final model were: age, gender, stage, number of chemotherapy cycles, previous chemotherapy, platinum-containing chemotherapy, taxanes-containing chemotherapy regimen, cytotoxic antibiotics-containing chemotherapy regimen, pyrimidine analogues-containing chemotherapy regimen, baseline neutrophyl count, baseline lymphocyte count, baseline monocyte count, baseline platelet count, baseline bilirrubin, baseline LDH, baseline GOT, baseline GPT, baseline albumin, baseline haemoglobin, G-CSF use within 72 h after chemotherapy.

CI, confidence interval; G-CSF, granulocyte colony-stimulating factors; GOT, glutamyl oxaloacetic transaminase; GPT, glutamyl pyruvic transaminase; LDH, lactate-dehydrogenase; OR, odds ratio.

Figure 3b shows the incidence of severe neutropenia and febrile neutropenia in patients receiving daily G-CSF (<5 or ≥5 days) and pegfilgrastim. Patients receiving at least 5 days of daily G-CSF displayed better outcomes than patients with less than 5 days.

### Chemotherapy dose reductions and delays and response rate

Patients who received a daily G-CSF suffered more chemotherapy dose reductions and delays and presented a lower response rate (Table 6). Overall, 51.7% and 59.3% of dose reductions and delays, respectively, were due to neutropenia. Other reasons (multiple-choice variable) were: non-haematological toxicity (which caused 19.7% of dose reductions and 7.4% of dose delays), thrombocytopenia (15.0% and 11.3% respectively), anaemia (10.3% and 10.6%), other causes (27.8% and 29.9%).

**Table 6:** Chemotherapy dose delays and reductions and response rate in patients who received daily G-CSF or pegfilgrastim

Outcome	Daily G-CSF (*n* = 211)	Pegfilgrastim (*n* = 180)	
	
	*n* (%)			*P*-value
Dose reductions, *n* (%)	78 (38.4)	53 (31.6)	0.116
Dose delays, *n* (%)	111 (54.7)	70 (41.7)	0.013
Chemotherapy dose intensity <85%, *n* (%)	82 (39.4)	52 (28.9)	0.030
Complete response, *n* (%)	33 (17.0)	46 (26.4)	0.028
Response (partial + complete), *n* (%)	80 (41.2)	92 (52.9)	0.009

G-CSF, granulocyte colony-stimulating factors.

Table 7 shows the multivariate model for predicting chemotherapy dose reductions or delays (defined as chemotherapy dose intensity <85%). The independent factors associated to chemotherapy dose intensity <85% were a high number of chemotherapy cycles, a baseline neutrophil count <1.5 × 10^9^/L and having received G-CSF as SP or reactive treatment, relative to patients with G-CSF as PP. Platinum and taxane-containing chemotherapy was associated to a lower probability of having chemotherapy dose intensity <85%. The type of G-CSF did not remain as a significant predictor.

**Table 7:** Independent predictors of chemotherapy dose intensity <85% in patients with solid tumours (excluding breast cancer) treated with G-CSF in the clinical practice (multivariate logistic regression model)

Predictors of chemotherapy dose intensity <85%	Odds ratio (OR)	95% CI for OR	*P*-value
Baseline neutrophil count <1.5 × 10^9^/L	2.80	1.18–6.64	0.020
Platinum and taxane-containing chemotherapy regimen	0.51	0.30–0.87	0.013
Number of chemotherapy cycles (for each additional cycle)	1.10	1.03–1.21	0.046
Prophylactic G-CSF use (*ref. primary prophylaxis*)			<0.0005*
Secondary prophylaxis	3.09	1.75–5.44	<0.0005
Treatment	3.65	2.09–6.38	<0.0005
Daily G-CSF (*ref. pegfilgrastim*)	1.20	0.75–2.0	0.409

*n* = 388.

* *P*-value indicates the overall effect of the variable in the model.

Other variables tested and not included in the final model were: age, gender, stage, intention of chemotherapy treatment, previous chemotherapy, platinum-containing chemotherapy, taxanes-containing chemotherapy regimen, cytotoxic antibiotics-containing chemotherapy regimen, pyrimidine analogues-containing chemotherapy regimen, mustard analogue-containing chemotherapy regimen, baseline lymphocyte count, baseline monocyte count, baseline platelet count, baseline bilirrubin, baseline LDH, baseline GOT, baseline GPT, baseline creatinine, baseline albumin, baseline haemoglobin, G-CSF use within 72 h after chemotherapy.

CI, confidence interval; G-CSF, granulocyte colony-stimulating factors; GOT, glutamyl oxaloacetic transaminase; GPT, glutamyl pyruvic transaminase; LDH, lactate-dehydrogenase; OR, odds ratio.

Regarding response to cancer therapy, the independent factors predicting a higher probability of response in the multivariate analysis (Table 8) were chemotherapy with curative intent after surgery (adjuvant CT), relative to chemotherapy for metastatic disease and absence of previous chemotherapy. Patients with lung, gastric or soft tissue cancer had less probability of achieving an objective response than patients with other types of tumour. Administration of G-CSF as SP or reactive treatment was also independently associated to a lower probability of response compared with PP. The type of G-CSF did not remain as a significant predictor.

**Table 8:** Independent predictors of response to cancer therapy (partial or complete) in patients with solid tumours (excluding breast cancer) treated with G-CSF in the clinical practice (multivariate logistic regression model)

Predictors of response	Odds ratio (OR)	95% CI for OR	*P*-value
Absence of prior CT	2.68	1.62–4.42	<0.0005
Intention of chemotherapy treatment *(ref. Chemotherapy for metastatic disease)*			0.055
Neoadjuvant CT	1.61	0.90–2.86	0.106
Adjuvant CT	1.89	1.09–3.33	0.024
Lung cancer (*ref. all other tumour types*)	0.56	0.33–0.93	0.027
Gastric cancer (*ref. all other tumour types*)	0.40	0.17–0.93	0.039
Soft tissue cancer (*ref. all other tumour types*)	0.21	0.05–0.86	0.030
Prophylactic G-CSF use (*ref. primary prophylaxis*)			0.006*
Secondary prophylaxis	0.47	0.27–0.81	0.006
Treatment	0.49	0.28–0.85	0.011
Daily G-CSF (*ref. non-daily*)	0.84	0.47–1.5	0.557

*n* = 365.

* *P*-value indicates the overall effect of the variable in the model.

Other variables tested and not included in the final model were: age, gender, stage, head and neck cancer, number of chemotherapy cycles, platinum-containing chemotherapy, taxanes-containing chemotherapy regimen, mustard analogues-containing chemotherapy regimen, cytotoxic antibiotics-containing chemotherapy regimen, pyrimidine analogues-containing chemotherapy regimen, baseline neutrophyl count, baseline lymphocyte count, baseline monocyte count, baseline platelet count, baseline bilirrubin, baseline LDH, baseline GOT, baseline GPT, baseline creatinine, baseline albumin, baseline haemoglobin, G-CSF use within 72 h after chemotherapy.

CI, confidence interval; G-CSF, granulocyte colony-stimulating factors; GOT, glutamyl oxaloacetic transaminase; GPT, glutamyl pyruvic transaminase; LDH, lactate-dehydrogenase; OR, odds ratio.

### Safety

The main adverse reactions to G-CSF were bone pain and astenia (Table 9). A higher incidence of bone and muscle pain was observed with daily G-CSF, compared with pegfilgrastim (6.2% vs. 1.7%, *P* = 0.025). No discontinuations due adverse reactions were reported. Forty-eight patients died during the retrospective follow-up period. The reasons of death were: disease progression (*n* = 26), cerebrovascular disease (*n* = 6), overall health deterioration (*n* = 3), respiratory failure (*n* = 3), ischaemic heart disease (*n* = 4), infectious complications during neutropenia (*n* = 2), cardiac decompensation (*n* = 1), traffic accident (*n* = 1), unknown (*n* = 2).

**Table 9:** Incidence of adverse drug reactions during chemotherapy administration (and up to 2 months later) in patients who received daily G-CSF or pegfilgrastim

	Daily G-CSF (*n* = 211)	Pegfilgrastim (*n* = 180)	
	
	*n* (%)		*P*-value
Any adverse drug reaction	20 (9.5)	11 (6.1)	0.219
Bone and muscle pain	13 (6.2)	3 (1.7)	0.025
Astenia	5 (2.4)	4 (2.2)	1.000
Fever	4 (1.9)	1 (0.6)	0.380
Dyspnea	2 (0.9)	2 (1.1)	1.000
Anorexia	1 (0.5)	2 (1.1)	0.597
Diarrhoea	1 (0.5)	2 (1.1)	0.597

G-CSF, granulocyte colony-stimulating factors.

## Discussion

Our study provides updated information on patterns of treatment and outcomes in patients with solid tumours (excluding breast cancer) treated with daily and non-daily G-CSFs in the Spanish oncology services. The two study cohorts were comparable with regard to disease severity, previous cancer treatments, clinical status at chemotherapy initiation and prescribed chemotherapy regimens, but patients who received a daily G-CSF were older and completed a higher number of chemotherapy cycles, which suggests a higher risk for neutropenia in this subgroup. Clearly, the non-daily G-CSF pegfilgrastim was more frequently used (almost double) for primary or secondary prophylaxis than filgrastim or lenograstim. After adjusting by all these possible confounders, the multivariate analysis showed an approximately 70% decrease in the probability of developing severe neutropenia in patients who received pegfilgrastim compared with patients who received a daily G-CSF. A trend toward less hospitalisation due to febrile neutropenia when pegfilgrastim is used was also observed after extensive adjustment, but probably the low rate of events did not allow to find significant differences. Better neutropenia-outcomes in patients with pegfilgrastim were translated into fewer chemotherapy dose delays and reductions, higher response rate, and less antibiotic use. However, the effect on chemotherapy dose intensity and response rate was probably due to an indirect association with G-CSF use as primary prophylaxis, since the type of G-CSF did not remain as an independent predictor of these two outcomes after the introduction of prophylactic G-CSF use into the equations.

The administration of primary prophylaxis from the first cycle, independently of the patients and chemotherapy characteristics, and of the type of G-CSF used, protected against severe neutropenia and febrile neutropenia development and was associated to higher chemotherapy dose intensity and response rate. Patients selected for primary prophylaxis had more advanced disease and received less number of chemotherapy cycles with more taxane-containing regimens than patients with secondary prophylaxis or treatment, which could have biased neutropenia outcomes, but the multivariate analyses was adjusted by all these factors. Thus, our results suggest that the benefits of primary prophylaxis on chemotherapy dose intensity and response rate in our two cohorts were directly due to the reduction in neutropenia incidence, which was the main cause of chemotherapy dose delays and reductions.

All these findings combined support that primary prophylaxis with pegfilgrastim effectively reduces the incidence of neutropenia and its related events and achieves the best outcomes in the clinical practice, with less than 5% of patients requiring hospitalisation due to this complication (Ozer *et al*. 2007). An integrated analysis of clinical trials and observational studies in breast cancer patients found similar results (von Minckwitz *et al*. 2009). Also other studies have reported better outcomes for pegfilgrastim versus filgrastim after adjusting by patient and chemotherapy characteristics (Morrison *et al*. 2007). Since no superiority of pegfilgrastim over filgrastim or lenograstim has been demonstrated in clinical trials (Holmes *et al*. 2002a; Grigg *et al*. 2003; Schippinger *et al*. 2006), we hypothesise that the incorrect administration of daily G-CSFs in current practice might explain the observed differences. The sustained duration of action of pegfilgrastim ensures an increase in neutrophil counts for as long as neutropenia is present (Shochat & Rom-Kedar 2008), but if daily G-CSF injections are withdrawn too soon, the efficacy is compromised (Shochat & Rom-Kedar 2008). In the present study, patients who received daily GCSF often initiated treatment later than recommended and received fewer days per cycle than demonstrated to be effective in randomised controlled trials, as found in previous observational studies (Koumakis *et al*. 1999; Scott *et al*. 2003; Weycker *et al*. 2006; Morrison *et al*. 2007; Almenar *et al*. 2009). Reduction of daily G-CSF treatment duration has been demonstrated to increase the incidence of febrile neutropenia (Scott *et al*. 2003; Marina *et al*. 2009) and hospitalisation (Weycker *et al*. 2006). There is also evidence that delaying G-CSF treatment initiation increases the depth and duration of ANC nadir and delays ANC recovery, as demonstrated by a phase II trial (Crawford *et al*. 1997) and increases the incidence and duration of febrile neutropenia (Koumakis *et al*. 1999). Thus, the suboptimal use of a daily G-CSF may represent an increased cost per patient compared with pegfilgrastim, as demonstrated by a recent pharmacoeconomic model in breast cancer patients at high risk of febrile neutropenia (Cámara *et al*. 2008).

Most of the factors significantly associated with severe neutropenia in our sample had been previously reported (Morrison *et al*. 2007; Pettengell *et al*. 2009). Chemotherapy dose delays and reductions were independently associated to a high number of chemotherapy cycles and a low baseline neutrophil count. Unexpectedly, non-platinum and taxane-containing chemotherapy was also associated with reduced chemotherapy delivery. It is possible that physicians prescribing regimens that include both platinum and taxanes are more aware of neutropenia complications and, thus, more prone to use G-CSF prophylaxis in a proper way. In fact, we observed that patients receiving taxanes had more G-CSF use as primary prophylaxis than patients with other chemotherapy regimens. Regarding response to cancer therapy, main predictors in the study sample were chemotherapy with curative intent before or after surgery and absence of previous chemotherapy. In addition, patients with lung, gastric or soft tissue cancer had less probability of achieving an objective response than patients with other types of tumour.

The incidence of adverse reactions was similar or even lower than that reported in clinical trials (Holmes *et al*. 2002a; Grigg *et al*. 2003; Schippinger *et al*. 2006). Pegfilgrastim was associated to a lower incidence of bone pain compared with daily G-CSF.

Our study has some limitations. The study cohort included a highly heterogeneous group of patients, with many tumour types at different stages who received distinct chemotherapy regimens. The efficacy of G-CSFs in solid tumours has been proven mainly in randomised trials including patients with breast cancer, sarcoma or small cell lung cancer (Crawford *et al*. 1991; Bui *et al*. 1995; Muhonen *et al*. 1996; Gatzemeier *et al*. 2000; Timmer-Bonte *et al*. 2005; Vogel *et al*. 2005), but data from other tumour types are scarce. Observational studies suggest that the effectiveness of G-CSFs is independent of tumour location and, in fact, current NCCN guidelines for use of myeloid growth factors are common to all malignancies (NCCN 2012). According to the developed prediction equations, the risk of neutropenia differs in solid malignancies mainly due to the characteristics of the chemotherapy regimen and to some patient-related factors (age above 65 years, prior FN, other infections …), but the tumour site by itself does not seem to be an independent predictor of FN risk (Lyman *et al*. 2005, 2011; NCCN 2012). We entered this variable in the multivariate analyses, but the limited sample size of many tumour types did not allow us to perform stratified analyses in each location. Other limitations are the observational design, that does not allow to exclude bias by indication, and the retrospective data collection. Although we have performed a multivariate analysis to adjust by possible confounders, we cannot exclude residual confounding due to non-tested variables. Thus, results must be interpreted with caution.

In conclusion, our data suggest that the use of pegfilgrastim compared with a daily G-CSF, and primary prophylaxis compared with secondary prophylaxis could decrease the incidence of neutropenia and its related events in patients with solid tumours undergoing chemotherapy. Most patients do not receive daily G-CSF as per label, which can compromise its efficacy.

## References

[b1] Aapro MS, Cameron DA, Pettengell R, Bohlius J, Crawford J, Ellis M, Kearney N, Lyman GH, Tjan-Heijnen VC, Walewski J, Weber DC, Zielinski C (2006). EORTC guidelines for the use of granulocyte-colony stimulating factor to reduce the incidence of chemotherapy-induced febrile neutropenia in adult patients with lymphomas and solid tumours. European Journal of Cancer.

[b2] Almenar D, Mayans J, Juan O, Bueno JM, Lopez JI, Frau A, Guinot M, Cerezuela P, Buscalla EG, Gasquet JA, Sanchez J (2009). Pegfilgrastim and daily granulocyte colony-stimulating factor: patterns of use and neutropenia-related outcomes in cancer patients in Spain – results of the LEARN Study. European Journal of Cancer Care.

[b3] Bishop MR, Tarantolo SR, Geller RB, Lynch JC, Bierman PJ, Pavletic ZS, Vose JM, Kruse S, Dix SP, Morris ME, Armitage JO, Kessinger A (2000). A randomized, double-blind trial of filgrastim (granulocyte colony-stimulating factor) versus placebo following allogeneic blood stem cell transplantation. Blood.

[b4] Bui BN, Chevallier B, Chevreau C, Krakowski I, Peny AM, Thyss A, Maugard-Louboutin C, Cupissol D, Fargeot P, Bonichon F (1995). Efficacy of lenograstim on hematologic tolerance to MAID chemotherapy in patients with advanced soft tissue sarcoma and consequences on treatment dose-intensity. Journal of Clinical Oncology.

[b5] Cámara JIM, Pousa AL, García EG, Llach XB, Surinyach NL, Vilela FS, Liu Z, Arocho R (2008). Evaluación económica del uso de pegfilgrastim frente a filgrastim en profilaxis primaria en pacientes con cáncer de mama con riesgo de padecer neutropenia febril en España. PharmacoEconomics.

[b8] Crawford J, Ozer H, Stoller R, Johnson D, Lyman G, Tabbara I, Kris M, Grous J, Picozzi V, Rausch G, Smith R, Gradishar W, Yahanda A, Vincent M, Stewart M, Glaspy J (1991). Reduction by granulocyte colony-stimulating factor of fever and neutropenia induced by chemotherapy in patients with small-cell lung cancer. The New England Journal of Medicine.

[b7] Crawford J, Kreisman H, Garewal H, Jones SE, Shoemaker D, Pupa MR, Armstrong S, Tomita D, Dziem G (1997). The impact of Filgrastim schedule variation on hematopoietic recovery post-chemotherapy. Annals of Oncology.

[b6] Crawford J, Green M, McGuire B, Shahin S (2005). A combined analysis of average relative dose intensity in the chemotherapy of solid tumors with pegfilgrastim or filgrastim support. Supportive Cancer Therapy.

[b9] Dale DC, Bonilla MA, Davis MW, Nakanishi AM, Hammond WP, Kurtzberg J, Wang W, Jakubowski A, Winton E, Lalezari P (1993). A randomized controlled phase III trial of recombinant human granulocyte colony-stimulating factor (filgrastim) for treatment of severe chronic neutropenia. Blood.

[b10] Eldar-Lissai A, Cosler LE, Culakova E, Lyman GH (2008). Economic analysis of prophylactic pegfilgrastim in adult cancer patients receiving chemotherapy. Value in Health.

[b11] Gabrilove JL (2006). An analysis of current neutropenia therapies, including pegfilgrastim. Clinical Cornerstone.

[b12] Gatzemeier U, Kleisbauer JP, Drings P, Kaukel E, Samaras N, Melo MJ, Cardenal F, Robinet G, Snijder RJ, Von Pawel J, Palisses R (2000). Lenograstim as support for ACE chemotherapy of small-cell lung cancer: a phase III, multicenter, randomized study. American Journal of Clinical Oncology.

[b13] Green MD, Koelbl H, Baselga J, Galid A, Guillem V, Gascon P, Siena S, Lalisang RI, Samonigg H, Clemens MR, Zani V, Liang BC, Renwick J, Piccart MJ (2003). A randomized double-blind multicenter phase III study of fixed-dose single-administration pegfilgrastim versus daily filgrastim in patients receiving myelosuppressive chemotherapy. Annals of Oncology.

[b14] Grigg A, Solal-Celigny P, Hoskin P, Taylor K, McMillan A, Forstpointner R, Bacon P, Renwick J, Hiddemann W (2003). Open-label, randomized study of pegfilgrastim vs. daily filgrastim as an adjunct to chemotherapy in elderly patients with non-Hodgkin's lymphoma. Leukemia and Lymphoma.

[b15] Holmes FA, Jones SE, O'shaughnessy J, Vukelja S, George T, Savin M, Richards D, Glaspy J, Meza L, Cohen G, Dhami M, Budman DR, Hackett J, Brassard M, Yang BB, Liang BC (2002a). Comparable efficacy and safety profiles of once-per-cycle pegfilgrastim and daily injection filgrastim in chemotherapy-induced neutropenia: a multicenter dose-finding study in women with breast cancer. Annals of Oncology.

[b16] Holmes FA, O'shaughnessy JA, Vukelja S, Jones SE, Shogan J, Savin M, Glaspy J, Moore M, Meza L, Wiznitzer I, Neumann TA, Hill LR, Liang BC (2002b). Blinded, randomized, multicenter study to evaluate single administration pegfilgrastim once per cycle versus daily filgrastim as an adjunct to chemotherapy in patients with high-risk stage II or stage III/IV breast cancer. Journal of Clinical Oncology.

[b17] Koumakis G, Vassilomanolakis M, Barbounis V, Hatzichristou E, Demiri S, Plataniotis G, Pamouktsoglou F, Efremidis AP (1999). Optimal timing (Preemptive versus supportive) of granulocyte colony-stimulating factor administration following high-dose cyclophosphamide. Oncology.

[b18] Kuderer NM, Dale DC, Crawford J, Cosler LE, Lyman GH (2006). Mortality, morbidity, and cost associated with febrile neutropenia in adult cancer patients. Cancer.

[b19] Kuderer NM, Dale DC, Crawford J, Lyman GH (2007). Impact of primary prophylaxis with granulocyte colony-stimulating factor on febrile neutropenia and mortality in adult cancer patients receiving chemotherapy: a systematic review. Journal of Clinical Oncology.

[b20] Lyman GH (2006). Risks and consequences of chemotherapy-induced neutropenia. Clinical Cornerstone.

[b22] Lyman GH, Lyman CH, Agboola O (2005). Risk models for predicting chemotherapy-induced neutropenia. The Oncologist.

[b21] Lyman GH, Kuderer NM, Crawford J, Wolff DA, Culakova E, Poniewierski MS, Dale DC (2011). Predicting individual risk of neutropenic complications in patients receiving cancer chemotherapy. Cancer.

[b23] Marangolo M, Bengala C, Conte PF, Danova M, Pronzato P, Rosti G, Sagrada P (2006). Dose and outcome: the hurdle of neutropenia (Review). Oncology Reports.

[b24] Marina J, Carabantes FJ, Escrivá S, Pernas S, Cantos B, Carañana V, Llorca C, Vázquez F, Ciruelos E, Menjón S (2009). Current practice of prophylaxis with granulocyte colony-stimulating factors for preventing chemotherapy-induced neutropenia in breast cancer patients in Spain. European Journal of Cancer Supplements.

[b25] Messori A, Trippoli S, Tendi E (1996). G-CSF for the prophylaxis of neutropenic fever in patients with small cell lung cancer receiving myelosuppressive antineoplastic chemotherapy: meta-analysis and pharmacoeconomic evaluation. Journal of Clinical Pharmacy and Therapeutics.

[b26] Morrison VA, Wong M, Hershman D, Campos LT, Ding B, Malin J (2007). Observational study of the prevalence of febrile neutropenia in patients who received filgrastim or pegfilgrastim associated with 3–4 week chemotherapy regimens in community oncology practices. Journal of Managed Care Pharmacy.

[b27] Muhonen T, Jantunen I, Pertovaara H, Voutilainen L, Maiche A, Blomqvist C, Pyrhonen S, Kellokumpu-Lehtinen P (1996). Prophylactic filgrastim (G-CSF) during mitomycin-C, mitoxantrone, and methotrexate (MMM) treatment for metastatic breast cancer. A randomized study. American Journal of Clinical Oncology.

[b28] NCCN (2012). http://www.nccn.org.

[b29] Ozer H, Mirtsching B, Rader M, Luedke S, Noga SJ, Ding B, Dreiling L (2007). Neutropenic events in community practices reduced by first and subsequent cycle pegfilgrastim use. The Oncologist.

[b30] Pettengell R, Bosly A, Szucs TD, Jackisch C, Leonard R, Paridaens R, Constenla M, Schwenkglenks M (2009). Multivariate analysis of febrile neutropenia occurrence in patients with non-Hodgkin lymphoma: data from the INC-EU Prospective Observational European Neutropenia Study. British Journal of Haematology.

[b31] Pinto L, Liu Z, Doan Q, Bernal M, Dubois R, Lyman G (2007). Comparison of pegfilgrastim with filgrastim on febrile neutropenia, grade IV neutropenia and bone pain: a meta-analysis of randomized controlled trials. Current Medical Research and Opinion.

[b33] Schippinger W, Holub R, Dandachi N, Bauernhofer T, Samonigg H (2006). Frequency of febrile neutropenia in breast cancer patients receiving epirubicin and docetaxel/paclitaxel with colony-stimulating growth factors: a comparison of filgrastim or lenograstim with pegfilgrastim. Oncology.

[b32] Scott SD, Chrischilles EA, Link BK, Delgado DJ, Fridman M, Stolshek BS (2003). Days of prophylactic filgrastim use to reduce febrile neutropenia in patients with non-Hodgkin's lymphoma treated with chemotherapy. Journal of Managed Care Pharmacy.

[b34] Shochat E, Rom-Kedar V (2008). Novel strategies for granulocyte colony-stimulating factor treatment of severe prolonged neutropenia suggested by mathematical modeling. Clinical Cancer Research.

[b35] Siena S, Piccart MJ, Holmes FA, Glaspy J, Hackett J, Renwick JJ (2003). A combined analysis of two pivotal randomized trials of a single dose of pegfilgrastim per chemotherapy cycle and daily Filgrastim in patients with stage II–IV breast cancer. Oncology Reports.

[b36] Timmer-Bonte JN, De Boo TM, Smit HJ, Biesma B, Wilschut FA, Cheragwandi SA, Termeer A, Hensing CA, Akkermans J, Adang EM, Bootsma GP, Tjan-Heijnen VC (2005). Prevention of chemotherapy-induced febrile neutropenia by prophylactic antibiotics plus or minus granulocyte colony-stimulating factor in small-cell lung cancer: a Dutch Randomized Phase III Study. Journal of Clinical Oncology.

[b37] Vogel CL, Wojtukiewicz MZ, Carroll RR, Tjulandin SA, Barajas-Figueroa LJ, Wiens BL, Neumann TA, Schwartzberg LS (2005). First and subsequent cycle use of pegfilgrastim prevents febrile neutropenia in patients with breast cancer: a multicenter, double-blind, placebo-controlled phase III study. Journal of Clinical Oncology.

[b38] Von Minckwitz G, Schwenkglenks M, Skacel T, Lyman GH, Pousa AL, Bacon P, Easton V, Aapro MS (2009). Febrile neutropenia and related complications in breast cancer patients receiving pegfilgrastim primary prophylaxis versus current practice neutropaenia management: results from an integrated analysis. European Journal of Cancer.

[b39] Weycker D, Hackett J, Edelsberg JS, Oster G, Glass AG (2006). Are shorter courses of filgrastim prophylaxis associated with increased risk of hospitalization?. The Annals of Pharmacotherapy.

